# Effect of Nutrient and Micronutrient Intake on Chylomicron Production and Postprandial Lipemia

**DOI:** 10.3390/nu11061299

**Published:** 2019-06-08

**Authors:** Charles Desmarchelier, Patrick Borel, Denis Lairon, Marie Maraninchi, René Valéro

**Affiliations:** 1 Aix-Marseille Université, Faculty of Medicine, 27 Boulevard Jean Moulin, 13385 Marseille, France; Charles.DESMARCHELIER@univ-amu.fr (C.D.); Patrick.BOREL@univ-amu.fr (P.B.); denis.lairon@orange.fr (D.L.); Marie.MARANINCHI@univ-amu.fr (M.M.); 2 C2VN (Center for Cardiovascular and Nutrition Research), Faculty of Medicine, 27 Boulevard Jean Moulin, 13385 Marseille, France; 3 INSERM, Faculty of Medicine, 27 Boulevard Jean Moulin, 13385 Marseille, France; 4 INRA, Faculty of Medicine, 27 Boulevard Jean Moulin, 13385 Marseille, France; 5 APHM (Assistance Publique-Hôpitaux de Marseille), CHU Conception, 147 Boulevard Baille, 13385 Marseille, France

**Keywords:** carbohydrates, cardiovascular disease, cholesterol, fibers, food structure, lipids, polyphenols, proteins, triglycerides, vitamins

## Abstract

Postprandial lipemia, which is one of the main characteristics of the atherogenic dyslipidemia with fasting plasma hypertriglyceridemia, low high-density lipoprotein cholesterol and an increase of small and dense low-density lipoproteins is now considered a causal risk factor for atherosclerotic cardiovascular disease and all-cause mortality. Postprandial lipemia, which is mainly related to the increase in chylomicron production, is frequently elevated in individuals at high cardiovascular risk such as obese or overweight patients, type 2 diabetic patients and subjects with a metabolic syndrome who share an insulin resistant state. It is now well known that chylomicron production and thus postprandial lipemia is highly regulated by many factors such as endogenous factors: circulating factors such as hormones or free fatty acids, genetic variants, circadian rhythms, or exogenous factors: food components, dietary supplements and prescription drugs. In this review, we focused on the effect of nutrients, micronutrients and phytochemicals but also on food structure on chylomicron production and postprandial lipemia.

## 1. Introduction

Cardiovascular diseases (CVD) are the leading cause of death in the world [1]. Atherogenic dyslipidemia (AD), which is mainly characterized by plasma fasting and postprandial hypertriglyceridemia (postprandial hyperlipemia), low high-density lipoprotein cholesterol (HDL-C) and an increase of small and dense low-density lipoproteins (LDL), is frequently seen in individuals at high cardiovascular risk such as obese or overweight patients, type 2 diabetic (T2D) patients and subjects with a metabolic syndrome who share an insulin resistant state [2,3]. The pathophysiology of the AD is widely explained by the blood accumulation of triglyceride-rich lipoproteins (TRL) synthesized by the liver (very low-density lipoproteins (VLDL)) [4] and the intestine (chylomicrons (CM)) [5]. This accumulation has been attributed to the overproduction of both VLDL and CM and to a defective TRL removal process [6,7]. Elevated fasting and postprandial blood TRL concentrations, which are mainly related to the increase in CM production, are now considered a causal risk factor for low-grade inflammation, atherosclerotic CVD and all-cause mortality [8]. It is now well known that CM production is highly regulated by many factors such as endogenous factors: circulating factors such as hormones or free fatty acids (FFA), genetic variants, circadian rhythms, or exogenous factors: food components, nutraceuticals and therapeutic interventions. In this review, we will focus on the effect of nutrients, micronutrients and phytochemicals but also on food structure on CM production and postprandial lipemia (TRL metabolism only) in humans [9].

## 2. Methodological Introduction

A large number of studies have assessed the effects of acute or chronic ingestion of meals containing different types of fat or other nutrients on postprandial lipemia but have yielded conflicting results. A number of potentially confounding factors reflecting the lack of standardization among studies could explain this: population, amount of fat, type of fat, amount and types of other nutrients, physicochemical composition of the meal and fatty acid (FA) or other nutrients, composition of habitual chronic food intake. Moreover, the measurement of postprandial plasma triglyceride (TG) response may provide only a limited evaluation of the true impact of meals and nutrients on postprandial lipoprotein metabolism. Studies have analyzed TG or retinyl-palmitate in a variety of sample types, including whole blood, plasma, serum, lipoproteins and their remnants, over a wide range from two up to 12 h postprandially [10]. Finally, qualitative and not only quantitative changes (size beyond the number of lipoproteins, lipidomic changes) have been described in several studies [11,12].

## 3. Effect of Dietary Lipids

### 3.1. Lipid Amount

A study performed in healthy men showed an increase in postprandial lipemia (plasma-TG peak concentration) following an 80 g fat meal compared to a 20 g fat meal with an intermediate result following a 40 g fat meal. A parallel elevation of glucose-dependent insulinotropic polypeptide (GIP) concentration and postheparin lipoprotein lipase (LPL) activity with a trend but no significant change in the increase of insulin response were seen following the 80 g fat meal compared to the 20 g fat meal [13]. Another study in healthy men individuals did not report any effect of a 15 g low-fat meal on postprandial lipemia compared to a nonfat meal and confirmed the dose-dependent increased postprandial serum-TG and CM-TG concentrations for moderate doses of fat per meal (30 to 50 g). The serum insulin response was significantly higher only following the 50 g fat meal compared to the nonfat and 15 g fat meal [14]. These results were confirmed in normal-weight and obese individuals with an increase in CM-TG concentration following a 40 g fat meal compared to a 10 g fat meal [15] and in obese boys with an increase in postprandial plasma-TG concentration following a 33 g fat meal compared to a 18 g fat meal. Glucagon-like-peptide-1 (GLP-1) concentration was significantly higher after the high-fat meal in the latter study [16]. Very high doses of fat (80 g and above) exaggerated postprandial serum-TG in healthy men [17]. This stepwise increase in the postprandial lipemia seen with the increase amount of fat intake suggests that the clearance capacity of the individuals is overloaded proportionally to the amount of fat assimilated. Moreover, consecutive meals containing fat appear to enhance postprandial lipemia [7,18].

### 3.2. Fatty Acid Composition

#### 3.2.1. Test Meal (Acute)

First, it should be remembered that dietary short- or medium-chain FA have limited effect on postprandial lipemia because they directly enter the general circulation via portal route instead of CM secretion. It is probably why studies using dairy fats, which contain significant amount of short- and medium-chain FA, as the only source of saturated fatty acids (SFA), generally report a lower postprandial TG response compared to other sources of SFA or other types of fats [7,19]. In healthy individuals, a recent randomized, cross-over, single-blinded design study, showed that a medium-chain SFA-rich meal (coconut biscuit) resulted in a significant lower postprandial whole blood TG response (concentration and net area under the curve (AUC)) compared to a short-chain SFA-rich meal (butter biscuit) and a long-chain SFA-rich meal (lard biscuit) despite identical fat and caloric content [20]. In healthy men, a study showed an increase in CM-TG concentration after an SFA-rich meal compared to an *n*-6 polyunsaturated fatty acid (PUFA)-rich meal but no difference with the monounsaturated fatty acid (MUFA)-rich meal [21]. The postprandial lipemic response to a SFA-rich meal was comparable to that of a *n*-6 PUFA-rich meal when consumed with *n*-3 PUFA in one study conducted in healthy individuals [22] whereas, the increase of the *n*-3 PUFA content of a SFA-rich meal fat meal did not acutely change postprandial TG concentration in another study performed in subjects with a metabolic syndrome [23]. In overweight men, a difference in the postprandial serum TG concentrations between an SFA-rich meal and an *n*-6 PUFA-rich meal was only seen in the late postprandial phase [24]. Four studies showed different effects of SFA compared to MUFA consumption on postprandial lipemia showing either an increase [25] or a decrease in healthy individuals [25,26] or in overweight and obese subjects [27], and one no change in healthy individuals [28]. In healthy individuals, another study showed a decrease in postprandial lipemia following a stearic acid-rich fat meal compared to other SFA (palmitic acid) or a high-oleic acid sunflower oil [29] but there was no difference between a high-oleic acid sunflower oil and a stearic acid-rich fat meal using cocoa butter [30]. Consumption of six different test meals rich in stearic, palmitic, palmitic plus myristic, oleic, elaidic or linoleic acids by healthy individuals resulted in a relatively lower postprandial lipemia response with long-chain SFA than did the intake of the unsaturated FA. The significant differences in LPL activities between groups did not explain the postprandial response that could be due to slower or less-efficient absorption of long-chain SFA [31]. Moreover, no difference in postprandial lipemia was observed following an oleate-rich meal (cis isomer) and an elaidate-rich meal (the trans isomer of oleic acid) [29]. Thus, most studies have shown that meals enriched with different proportion of SFA, MUFA or *n*-6 PUFA do not elicit marked differences in postprandial lipemia [7,32,33]. In healthy individuals, an *n*-3 PUFA-rich fat meal (fish oil) lowered postprandial lipemia compared to an SFA-rich fat meal (palm and coconut oils) [34]. However, two other studies did not show a difference in the incremental area under the curve (iAUC) plasma-TG after an SFA-rich meal compared to an *n*-3 PUFA-rich meal [28,35].

#### 3.2.2. The Habitual Diet (Chronic)

Postprandial lipemia may be influenced by the habitual diet [7,19]. A short-term consumption (25 days) of isocaloric diets rich in SFA, *n*-6 PUFA or *n*-3 PUFA in healthy individuals resulted in greater TG and CM concentrations following SFA compared to *n*-3 PUFA, with intermediate concentrations with *n*-6 PUFA. CM from subjects on *n*-3 and *n*-6 PUFA diets were more susceptible to lipolysis in vitro [36]. After 15 and 29 days of dietary intervention (SFA-rich or *n*-6 PUFA-rich diet in healthy young men), postprandial response analysis suggested no change in the clearance of CM remnants but a prolonged accumulation of VLDL in individuals fed with the SFA-rich diet [37]. Two other short-term studies (six or four weeks) showed that *n*-3 PUFA supplementation (2.7 g/day or 4 g/day) in healthy subjects resulted in a significant reduction in postprandial TG compared with the control diet without supplementation [38,39]. In the second study, the supplementation with *n*-3 PUFA suppressed the increase in TG content in CM as well as in VLDL [39]. In contrast, consumption of a low-fat diet with *n*-3 PUFA (fish oil) supplementation for 16 weeks in healthy individuals led to a significant increase in postprandial TG concentration following a fat-rich test meal compared to chronic consumption of a low-fat diet alone [40] and another study did not show an effect of a six month *n*-3 PUFA supplementation in fasting or postprandial lipids compared to *n*-6 PUFA supplementation in moderately hyperlipidemic subjects [41]. In healthy individuals, an eight-week study of diet rich in SFA or MUFA (olive oil) showed a significant reduction in plasma total- and LDL-cholesterol concentrations but a higher postprandial plasma TG and TRL-apoB-48 concentrations with the MUFA diet [42]. In contrast, a 16-week moderate-MUFA diet or high-MUFA diet following an eight-week SFA-rich diet (reference diet) in healthy individuals resulted in the reduction of postprandial apoB-48 response without change in plasma TG concentration, suggesting that the CM formed carry larger amounts of dietary lipids per particle [43]. Similarly, a three-month SFA-rich diet compared to a MUFA-rich diet in healthy individuals did not show a difference in postprandial TG concentration but a reduction in both groups receiving a *n*-3 PUFA supplementation (3.6 g/day) versus placebo [44]. In this study, neither type of diet nor *n*-3 PUFA supplementation affected serum LDL size, but this parameter was measured in fasting state. Furthermore, in a cohort of 1048 subjects, hypertriglyceridemic (fasting serum TG > 150 mg/dL) participants had higher number of LDL particles, higher concentrations of small LDL particles and lower large LDL particles in baseline compared to normotriglyceridemic (fasting serum TG ≤ 150 mg/dL) participants. Following a high-fat meal challenge, both groups displayed similar patterns of change in LDL particle size concentrations with a small decrease in total LDL particle number, an increase in large LDL particle concentration, a decrease in small LDL particle concentration and no change in LDL particle size [45]. In another study, healthy individuals consumed three different diets for four weeks: a Western diet (38% fat of which 22% SFA), Mediterranean diet (38% fat, 24% MUFA) and a high carbohydrates diet with α-linolenic acid (ALA) (< 30% fat of which 8% PUFA). Consumption of the Mediterranean diet led to a decrease in the postprandial number of TRL compared with the other meals and also an increase in TRL particle size compared to the high carbohydrates with α-linolenic acid diet [46]. In the Medi-Rivage intervention study, a postprandial test was performed in individuals after either a three-month low fat or a Mediterranean-type diet (with SFA intake reduced by about half whereas MUFA increased). The consumption of the Mediterranean diet only lowered fasting TG concentration and both diets reduced TG and apoB-48 levels 5 h after the test meal. The overall 5 h postprandial apoB-48 response (AUC and iAUC) was lowered after both diets but this effect was more marked after the Mediterranean diet intervention [47].

A two-week trans FA-rich diet compared to a MUFA-rich diet (oleate) in healthy individuals did not show a difference in postprandial TG after a test meal [48].

Concerning the mechanisms, the reduction of postprandial lipemia following *n*-3 PUFA supplementation could be due to a decrease in CM synthesis/secretion and/or an increase in clearance. Several studies have shown an increase in LPL activity [44,49,50,51] and hepatic lipase activity [51] following supplementation with 3–5 g/day *n*-3 long-chain PUFA suggesting an effect on CM clearance. On the other hand, some studies are more in favor of an effect on CM production [52,53]. It is important not to forget that the reducing effect of *n*-3 PUFA on VLDL production [53,54,55] could also influence postprandial lipemia by the major link with CM metabolism. A lipoprotein kinetic study has examined the effect of the addition of *n*-3 FA ethyl esters (4 g/day: 46% eicosapentaenoic acid (EPA) and 38% docosahexaenoic acid (DHA)) to a weight-loss program for 12 weeks on postprandial apoB-48 kinetics in obese subjects after ingestion of an oral load. Compared with weight loss alone, weight loss plus *n*-3 supplementation significantly decreased fasting TG, apoB-48 concentrations, postprandial TG and apoB-48 total AUCs as well as postprandial TG iAUCs. This improvement of the postprandial profile was due to a decreased apoB-48 secretion in the basal state in the *n*-3 supplementation group without a significant effect during the postprandial period (3–6 hours) and no change in the clearance rate compared to the weight loss alone group [56]. A crossover study (but without a lipoprotein kinetic analysis) conducted by the same team showed a significant improvement of the postprandial lipemia after a fat load in a group of patients with familial hypercholesterolemia with *n*-3 PUFA supplementation (8 weeks; 4 g/day: 46% eicosapentaenoic acid and 38% docosahexaenoic acid) compared to no supplementation [57]. A review mainly focused on stable isotope tracer methodologies and compartmental modeling studies examined the mechanisms of action of dietary FA on lipoprotein metabolism [58]. Concerning n-3 PUFA, their effect on TG concentration reduction can be explained by several mechanisms: inhibition of diacylglycerol acyltransferase, FA synthase and acetyl CoA carboxylase enzymes; increase of FA β-oxydation via a peroxisome proliferator-activated receptor (PPAR) mediated pathway; inhibition of de novo lipogenesis by suppressing transcription of sterol regulatory element-binding protein-1c (SREBP-1c) gene; degradation of newly synthesized apolipoprotein B by stimulating the post-endoplasmic reticulum presecretory proteolysis pathway. In vivo studies have confirmed that *n*-3 PUFA decrease the pool size (PS), the production rate (PR) of TRL-apoB-48 and VLDL-apoB-100, the fractional catabolic rate (FCR) of TRL-apoB-48 and increase the FCR of VLDL-apoB-100. Regarding *n*-6 PUFA, one lipoprotein kinetic study has shown after three weeks of *n*-6 PUFA supplementation a decrease in the PS of VLDL-apoB-100 due to an increase in FCR compared to a medium-chain FA supplementation. This effect could be due to an up-regulation of LPL activity and hepatic uptake of VLDL consequently to PPAR-activation by *n*-6 PUFA [59]. Lipoprotein kinetic studies on the impact of SFA on lipoprotein metabolism are lacking. One study did not show any effect of a four-week supplementation of medium-chain TG on TRL-apoB-48 and VLDL-apoB-100 metabolism in obese, insulin-resistant men [60]. For MUFA, one study conducted in twelve adults, has shown a decrease in PS and PR and an increase in FCR of VLDL-apoB-100 after a three-week MUFA-rich diet compared to a carbohydrate-rich diet [61]. Only one study has examined the impact of trans-FA on lipoprotein metabolism with no effect, in postmenopausal hypercholesterolemic women, on TRL-apoB-48 or TRL-apoB-100 metabolism [62].

#### 3.2.3. Clinical Trials and Recommendations

A position paper from an international lipid expert panel concluded that *n*-3 EPA and DHA could be used efficiently as dietary supplements to reduce plasma TG (by 18–25%) whereas, their effects on LDL-C and HDL-C were clinically insignificant [63].

Despite the potential positive effect of *n*-3 PUFA on fasting TG and postprandial lipemia, two meta-analyses of randomized controlled trial, including a recent one (77,917 subjects; EPA supplementation doses between 226 and 1800 mg/day; mean follow-up: 4.4 years), did not show any effect of *n*-3 PUFA supplementation on mortality and cardiovascular events [64,65]. The most recent and larger meta-analysis of randomized controlled trial (112,059 subjects; 12 to 72 months duration; *n*-3 PUFA doses ranged from 0.5 g/day to > 5 g/day including EPA and DHA or ALA by supplementation or enriched food or dietary advice compared to placebo or usual diet) showed that increasing EPA and DHA has little or no effect on mortality or cardiovascular health and that low-quality evidence suggested ALA may slightly reduce cardiovascular disease and arrhythmia risk [66].

However, a recent randomized, double-blind, placebo-controlled trial (REDUCE-IT) involving 8179 patients with established cardiovascular disease or with diabetes and other risk factors, who had been receiving statin therapy and who had a fasting TG level of 135 to 499 mg/dL and a LDL-C level of 41 to 100 mg/dL with a median follow-up of 4.9 years, showed that a high-dose treatment of 4 g/d of EPA is accompanied by a significant decrease of 25% in the primary endpoint (cardiovascular death, non-fatal myocardial infarction, non-fatal stroke, coronary revascularization, unstable angina), of 20% in cardiovascular mortality, of 28% in fatal and non-fatal strokes, and a non-significant decrease of 13% in total mortality. More patients were hospitalized for cardiac arrhythmias in the EPA group [67].

Regarding dietary fat intake, the European guidelines on cardiovascular disease prevention in clinical practice advise: SFA to account for < 10% of total energy intake and should be reduced by an increase in PUFA; trans unsaturated FA as little as possible with preferably no intake from processed food and < 1% of total energy intake from natural origin; fish 1–2 servings per week, one of which to be oily fish; 30 g unsalted nuts per day [68]. The recent American guidelines on the primary prevention of cardiovascular disease recommend: the replacement of SFA with dietary MUFA and PUFA; the intake of trans FA should be avoided: a diet containing reduced amounts of cholesterol [69].

The European guidelines for the management of dyslipidemias recommend reducing TRL levels: to use supplements *n*-3 PUFA and replace SFA with MUFA or PUFA [70].

In line with the most recent meta-analysis, supplemental long-chain *n*-3 PUFA are probably not useful for preventing or treating cardiovascular disease, although they can help to reduce serum TG and raise HDL a little [66].

### 3.3. Dietary Cholesterol

The data available on the impact of dietary cholesterol on postprandial lipemia are limited. In healthy individuals, one single meal containing 0 or 140 mg cholesterol with a fixed amount of fat (45 g) elicited comparable postprandial lipemia whereas important doses of dietary cholesterol (280 or 710 mg) significantly increased postprandial lipemia compared to the lower amount [71]. A cholesterol-rich meal (1 g) compared to a cholesterol-free meal elicited increased postprandial CM-cholesterol and CM-TG in T2D patients and in matched non-diabetic control subjects. The increase in the postprandial VLDL-apoB-48 concentration was significantly higher in the diabetic patients (10-fold) compared to control individuals (three-fold) but the postprandial VLDL-apoB-100 concentration was not affected by dietary cholesterol, suggesting that intestinal CM production rather than their clearance explained these results [72]. However, chronic consumption (eight weeks) of diets varying in their amount of cholesterol (from 128 to 858 mg/day) by healthy individuals had no effect on postprandial lipemia [73].

To summarize, it appears that postprandial lipemia increases dose-dependently with the amount of dietary fat or cholesterol (at least above 15–20 g of fat and 140 mg of cholesterol) after a single meal. Acute test meals enriched with SFA, MUFA or PUFA do not generally elicit markedly different postprandial lipemia. Despite conflicting results, habitual diet studies show postprandial lipidemic responses to be in the order SFA > MUFA > *n*-6 PUFA > *n*-3 PUFA.

## 4. Effect of Dietary Carbohydrates

### 4.1. Effect of Chronic Consumption of Dietary Carbohydrates

High carbohydrate diets and especially highly digestible carbohydrate enriched diets have commonly been shown to alter lipid postprandial metabolism and to increase fasting plasma TG as a result of both intestinal CM and hepatic VLDL TRL and their remnants accumulation [7]. In 2 groups of normolipidemic and moderately hypertriglyceridemic subjects, Parks et al. compared the effects of two isoenergetic diets: a control (35% fat) diet for one week followed by a low-fat (15% fat) and high-carbohydrate diet for 5 weeks. Low fat/high-carbohydrate diet resulted in increased fasting TG concentration and decreased VLDL-TG clearance rate in both groups and increased fasting TRL-apoB-48 and TRL-apoB-100 concentrations in the hypertriglyceridemic group [74].

The monosaccharides have been extensively studied. As described by Livesey et al. in a meta-analysis, effect of fructose consumption on lipid profile was different depending on daily ingested amount: significant effect on postprandial TG was not evident unless > 50 g fructose/day was consumed, and no significant effect was seen for fasting TG with intakes of ≤ 100 g fructose/day [75]. In 66 overweight or obese men, the consumption of fructose sweetened beverages containing 75 g fructose per day for 12 weeks while continuing usual lifestyle and diet increased significantly fasting plasma TG and postprandial TG (AUC and iAUC) response after a mixed meal but did not impair glycemic control or incretin hormone responses during oral glucose or mixed meal challenge [76]. In contrast, another meta-analysis on the replacement of glucose or sucrose in foods or beverages by fructose (11 trials; length from 2 to 10 weeks; doses of fructose between 40 and 150 g/day) found no difference in fasting TG when fructose replaced glucose but a slight significant reduction when fructose replaced sucrose [77]. In a meta-analysis of 10-week to 26-week randomized trials of sugar-containing soft drinks, Bray concluded that plasma TG increase was due to fructose rather than glucose in sugar-containing soft drinks [78]. However, in a randomized control clinical study, Campos et al. showed in overweight subjects that substitution of high sugar-sweetened beverages providing large amounts of mono- or di-saccharides by artificially sweetened beverages during 12 weeks did not decrease postprandial TG despite of lower energy and fructose content of the meals [79]. Stanhope et al. showed, in overweight and obese subjects, that the consumption (eight weeks) of fructose-sweetened beverages significantly lowered glucose and insulin postmeal peaks and the AUC compared with the baseline diet (energy balanced diet containing 55% of energy as complex carbohydrates for 2 weeks) and with the consumption of glucose-sweetened beverages. Only fructose sweetened beverages diet consumption resulted in increased postprandial TG suggesting that the specific effect of fructose, but not of glucose and insulin excursions, contribute to the adverse effects of consuming sugar-sweetened beverages on lipids and insulin sensitivity [80]. Glucose supplementation had no effect on postprandial TG response as confirmed in 2 other studies of the same authors in young men and women [81] or in overweight or obese subjects [72] but a specific increase in fasting plasma TG concentration [82]. Despland et al. found a slightly decrease in postprandial blood glucose but no difference in postprandial TG plasma nor in hepatic insulin sensitivity in eight healthy male consuming a diet containing 25% energy as honey or pure fructose-glucose compared to an isocaloric starch diet [83]. Taken together, these studies suggest that isocaloric inclusion of fructose in mixed meal has inconsistent effects on postprandial TG despite its greater stimulation of de novo lipogenesis than other monosaccharides such as glucose [84]. However, in a systematic review and meta-analysis of controlled feeding trials, Wang et al. showed no significant postprandial TG increase when fructose was exchanged isocalorically for other carbohydrates in the diet but a significant postprandial TG raising-effect of fructose in studies in which fructose supplemented the background diet with excess energy from high-dose fructose compared with the background diet alone (without the excess energy) [85].

### 4.2. Effect of Acute Consumption of Dietary Carbohydrates

Both amount and nature of carbohydrates in a meal may alter postprandial lipid metabolism.

In healthy individuals, a high-fat/low-carbohydrate meal yielded a postprandial TG iAUC increase and an apoB-48 plasma iAUC reduction compared to a low-fat/high carbohydrate meal. This suggests difference in size and composition of CM depending on the meal composition [86]. In healthy individuals, the addition of 50 g or 100 g oral glucose to a fatty test meal diminished postprandial lipemia in a dose dependent manner compared to the meal containing fat alone. This effect was not due to increased clearance of TG from the circulation but appeared to reflect delayed gastric emptying and decreased hepatic secretion of TG. Starch ingestion had no discernible effect on postprandial lipemia [87]. Likewise, the addition of 75 g oral glucose to an oral fat meal delayed the gastric emptying and postponed the CM response compared to the fat meal in healthy individuals. The postprandial iAUC of serum TG and VLDL-TG were reduced but the CM-TG iAUC remained unchanged. The postprandial reduction of VLDL-TG iAUC may be due to the pronounced FFA depression during the glucose-induced rise in insulin [88]. In contrast, in healthy individuals, adding oral fructose as a monosaccharide [89] or as a disaccharide in sucrose [90] to an oral fat load led to an increase in postprandial lipemia. 

In healthy individuals, physiological ranges of postprandial hyperglycemia and hyperinsulinemia as generated by starchy foods (white bread, pasta, beans) did not induce noticeable alterations in the overall postprandial TG response but delays and exacerbates postprandial accumulation of CM-apoB-48 in plasma [91]. Likewise, in healthy individuals, the intake of a standard fat dose meal (0.5 g/kg body weight) accompanied by either low-carbohydrate meal (17 g as lactose) or a high-carbohydrate meal (136 g of which 60 g was sucrose) did not show a difference in plasma TG or TRL-TG postprandial concentrations but a biphasic plasma TG response seen with the high-carbohydrate meal largely reflected the TRL-TG or CM fraction, which would tend to suggest a biphasic pattern of absorption. Higher insulin and GIP responses were seen with the high-carbohydrate meal [92]. Moreover, in obese, insulin-resistant subjects, the consumption of a high-glycemic index mixed meal, compared with a low-glycemic index one, increased the postprandial rise in plasma insulin and the accumulation of TRL-apoB-48 and TRL-apoB-100 thus increasing postprandial TG concentration as well as modifying the kinetics of peak occurrence. Thus, adding various digestible carbohydrates to a test meal can elicit a biphasic response of postprandial lipemia [93].

Besides the effects of dietary carbohydrates on hepatic VLDL metabolism [74,94], their effects on intestinal CM metabolism remain to be clarified, but have been reviewed in a recent paper pointing out the intestine as a contributor to carbohydrate-induced hyperlipidemia [95]. Oral fructose in a mixed meal can stimulate hepatic but also intestinal de novo lipogenesis, thereby increasing TG availability for CM and VLDL synthesis. This mechanism was increased for CM but not VLDL when glucose was added to the meal with a concomitant decrease in fructose oxidation and gluconeogenesis from fructose, suggesting the addition of glucose to the meal committed more fructose towards intestinal de novo lipogenesis [96]. In a lipoprotein kinetic study performed in healthy individuals, TRL-apoB-48 (CM) and TRL-apoB-100 (VLDL) metabolism was assessed after intraduodenal infusion (to avoid change in gastric emptying) of intralipid plus saline or glucose or fructose under pancreatic clamp conditions. Glucose markedly stimulated CM-PR with a moderate increase in CM-FCR resulting in net elevation of CM concentration but no effect on VLDL metabolism. Fructose significantly stimulated CM-PR and VLDL-PR but no effect on FCR [97]. The same team performed another lipoprotein kinetic study in healthy individuals to assess the effect of intravenous infusion of either 20% glucose or normal saline as control in a constant fed state. Compared with saline infusion; glucose infusion induced both hyperglycemia and hyperinsulinemia (despite pancreatic clamp conditions), FFA decrease and increased plasma TG, CM concentrations and CM-PR without affecting CM-FCR or VLDL metabolism [98]. Several studies have shown that a fructose-rich diet induced less insulin secretion than a glucose-rich diet, which could explain the lower postprandial LPL activity and the reduction in TG clearance after fructose compared to glucose [99].

At a cellular level, both luminal and basolateral glucose enhanced CM secretion with a greater effect of luminal glucose and a greater effect of luminal glucose than fructose [95]. In Caco-2/15 cells, basolateral exposure to glucose increased apical cholesterol uptake with increased expression of Niemann-Pick C1-like 1. This elevation of cholesterol uptake was associated with an increase in the transcription factors SREBP-2, carbohydrate-responsive element-binding protein (ChREBP) and liver X receptor (LXR)-β along with a fall in retinoid X receptor (RXR)-α [100]. Moreover, in Caco-2 cells, the incubation with glucose or fructose increased expression and protein abundance of microsomal triglyceride transfer protein (MTP) and fructose, but not glucose, activated SREBP-1 and ChREBP. These results show the link between carbohydrate and lipid pathways and suggest that these monosaccharides may play a role in enhancing TG synthesis and CM assembly [95]. Furthermore, several studies have shown that oral glucose compared to oral water can mobilize TG stored in cytosolic lipid droplets of enterocytes. This storage of droplets could contribute to CM appearance up to 16 h after the last meal. Moreover, the contractile activity of mesenteric lymphatics able to activate the secretion of extracellular CM, that reside between enterocytes, in lamina propria, lacteals and the mesenteric lymphatic system, was reduced with chronic high-fat and high-fructose feeding in rats [95].

### 4.3. Recommendations

Regarding dietary carbohydrate intake, the European guidelines on cardiovascular disease prevention in clinical practice advise: sugar-sweetened soft drinks must be discouraged [68]. The recent American guidelines on the primary prevention of cardiovascular disease recommend: to minimize the intake of refined carbohydrates and sweetened beverages [69]. 

The European guidelines for the management of dyslipidemias recommend reducing TRL levels: to reduce total amount of dietary carbohydrate and reduce intake of mono-disaccharides with a higher magnitude of the effect than the replacement of SFA with MUFA and PUFA. To illustrate this point, with a habitual fructose consumption between 15% and 20% of the total energy intake, plasma TG increase as much as 30–40% [70].

To summarize, it appears that postprandial lipemia increases more markedly with fructose than with glucose added to a single meal and in relation with the glycemic index of the carbohydrates. Acutely, a biphasic postprandial lipidemic response was described depending on the glycemic index of the carbohydrates. The chronic hypercaloric intake of fructose shows consistent results enhancing postprandial lipemia, but despite numerous studies, isocaloric chronic consumption of carbohydrates (fructose, glucose or starch) in mixed meals has led to discrepancies resulting in unclear divergent effects increasing or decreasing postprandial lipemia.

## 5. Effect of Dietary Proteins 

In recent years, several studies have investigated the effect of protein quantity and quality on postprandial lipemia. The main mechanisms by which proteins have been hypothesized to affect postprandial lipid concentrations are through their slowing down of gastric emptying [101] and their potent effect on insulin release, notably via increased incretin secretion, i.e., GIP and GLP-1 [102,103]. Insulin is a well-known activator of LPL [104] but an increase in postprandial insulin also inhibits hormone-sensitive lipase and thereby suppresses the release of FFA from adipose tissue [105], which could limit the lipotoxicity associated with elevated FFA concentrations [106].

### 5.1. Effect of Acute Addition of Dietary Proteins

Early work by Cohen et al. showed that the addition of 23 g casein to a meal containing 40 g fat did not have any effect on postprandial lipemia in a group of 15 healthy adults [107]. In a group of 24 healthy adults, the addition of 50 g sodium caseinate to a fat meal (1 g fat/kg body weight as whipping cream) did not have any effect on the AUC of postprandial serum-, CM- or VLDL-TG concentrations, although a delay in serum-, CM- and VLDL-TG peak concentrations was observed, without any difference in gastric emptying rates. However, addition of casein led to a 20% reduction in FFA concentration over 8 h, together with a 30% increase in insulin release [108]. In contrast, in a group of 16 healthy adults, the addition of 50 g sodium caseinate or soy protein to a fat meal (1 g fat/kg body weight as whipping cream) led to a decrease in postprandial TG concentration (significant decrease at early time points but AUC was not calculated), with a 1 h delay in the peak time, together with a decrease in FFA associated with an increase in insulin secretion [109]. 

In a group of 11 patients with well-controlled T2D, the addition of 45 g casein to a control meal, consisting of energy-free soup with 80 g of fat, did not affect the postprandial TG or HDL response. However, when casein was added to the control meal plus 45 g carbohydrates (as white bread), it suppressed the increased postprandial TG concentration observed after the control meal plus carbohydrates alone, with an increase in insulin, glucagon and GIP release and a decrease in FFA concentration [110].

### 5.2. Effect of Dietary Protein Type

Mortensen et al. compared the acute effect of protein type on postprandial lipemia by providing 12 patients with T2D a test meal containing 100 g butter and 45 g carbohydrates in combination with 45 g casein, whey, cod, or gluten [111]. Compared to other sources of proteins, whey led to a decrease in the AUC of postprandial TG concentration after 360 min (−27% to −31%), in both plasma and CM-rich fraction, suggesting a concomitant lower production of CM, as illustrated by a lower retinyl palmitate concentration in the CM-rich fraction and a decrease in FFA secretion. No differences in insulin, glucagon and incretin concentrations or gastric emptying were observed. Using the same setting in 11 obese non-diabetic patients, the same group observed a significant lowering effect of whey proteins on postprandial plasma TG, notably in the CM-rich fraction, with an increase in insulin and glucagon secretion compared to cod and gluten but not compared to casein [112]. In a group of 20 overweight or obese postmenopausal women, the addition of 45 g whey to a breakfast meal significantly decreased postprandial TG concentrations as well as the exposure to smaller TG-enriched CM particles, as reflected by a decrease in the AUC of the TG:apoB-48 ratio, compared to the addition of 45 g glucose or casein to the same meal (−21% and −27%, respectively) [113]. In 11 obese non-diabetic subjects, Homer-Jenssen et al. did not observe any difference between the addition of 45 g of four whey fractions (alpha-lactalbumin, whey isolate, caseino-glycomacro-peptide and whey hydrolysate) to a high-fat meal on postprandial TG, insulin, glucagon or incretin concentrations although whey hydrolysate led to a smaller decrease in postprandial FFA production compared to the other proteins [114]. No difference in postprandial TG concentration was observed in a similar setting in 12 T2D subjects [115].

Mariotti et al. provided 10 healthy overweight men a high-fat meal plus 45 g casein, whey protein, or α-lactalbumin-enriched whey protein and observed a lower increase in postprandial plasma TG concentration following the meal that provided casein (AUC decreased by 22%), with no effect on FFA or insulin. As supported by their in vitro observations at pH values similar to those observed in the stomach during digestion, the authors hypothesized that this difference was due to the low solubility of casein at low pH leading to potential phase separation in the stomach, hence slowing down the digestion and absorption of fat [116]. These results are in disagreement with those of Mortensen et al. and Pal et al. Mariotti et al. put forward that they studied healthy individuals and that the components of the meals they used were pre-mixed, therefore allowing more interaction between the nutrients. When providing a pre-meal consisting of 17.6 g proteins (whey, casein or gluten) 15 to 30 min before a fat-rich meal to 16 subjects with metabolic syndrome, Bjornshave et al. did not observe any difference with regard to postprandial TG or FFA concentrations [117]. The authors hypothesized that the lower protein dose used compared to Mariotti et al. may explain the discrepancy between the results of the two studies. The same group also provided 12 matched subjects with and without T2D with 17.6 g whey proteins 15 min before a fat-rich meal or during the main meal. Although the whey protein pre-meal led to an increase in insulin, glucagon and GIP concentrations in both groups of subjects and a decrease in gastric emptying rates, the authors did not observe any effect on postprandial TG, apoB-48 or FFA concentrations [118].

### 5.3. Effect of Chronic Addition of Dietary Proteins

Mamo et al. investigated the long-term effect of a diet enriched in proteins from lean red meat, in place of carbohydrates, on postprandial lipemia [119]. Twenty moderately hypertriglyceridemic but otherwise healthy individuals consumed for six weeks two isocaloric diets (14%, 53% and 30% energy from proteins, carbohydrates and fats respectively vs. 25%, 30% and 35%) and then received a fat tolerance test meal. The protein-enriched diet led to a decrease in CM production, with a lower postprandial apoB-48 concentration, but no difference in fasting plasma or postprandial lipemia was observed. In a group of 52 patients with abdominal obesity, Bohl et al. did not observe any long-term effect of the addition of 60 g whey compared with casein to a high-fat test meal consumed daily for 12 weeks on postprandial TG or FFA concentrations but whey addition led to a decrease in CM production compared to casein addition, with a lower postprandial apoB-48 concentration [120].

To summarize, although the effects of dietary proteins on gastric emptying and FFA release seem well established, their effect on postprandial TG concentrations remains unclear. Nonetheless, at least three studies have pointed at a greater decrease in postprandial TG caused by the acute consumption of whey proteins compared to casein, the two most studied protein sources. Of note, one study has observed an opposite effect but it was the only one where food components were pre-mixed, and therefore not physically separated from the fat source, thereby underlining the potential effect of food structure on postprandial TG concentrations, as further discussed in chapter eight of the present review. Long-term studies that investigated dietary protein consumption suggest a possible decreasing effect on CM production, in particular by whey proteins.

## 6. Effect of Dietary Fibers

The effect of dietary fibers on postprandial lipemia has previously been reviewed [7,121] showing that some sources of fibers, particularly soluble fibers, at the level of 4–10 g/meal, can decrease postprandial TG and cholesterol concentrations following a mixed meal. Several interrelated mechanisms have been put forward, including slowed gastric emptying, alteration of TG hydrolysis, through increased viscosity decreasing the rate of hydrolysis or inhibition of pancreatic lipase activity, alteration of mixed micelle formation, and possibly that of intestinal secretion of CM [122] but also modification of insulin secretion [123].

### 6.1. Effect of Acute Consumption of Dietary Fibers

Cara et al. showed that enrichment of a high-fat meal with fibers from cereals, i.e., 10 g oat bran, wheat fibers or 4.2 g wheat germ, led to a decreased postprandial serum TG concentration in 6 healthy male adults compared to a low-fiber control meal, while only wheat fibers led to a decrease in postprandial CM-TG concentration. Rice bran did not have any effect on postprandial TG concentration. All fiber sources led to a decrease in postprandial CM-cholesterol concentration [124]. In another study involving six ileostomized subjects, oat bran added to a test meal (43.8 g fat) was shown to elicit a 37% reduction in postprandial CM-TG concentration and a 43% reduction in postprandial CM-cholesterol concentration compared to a low fiber test meal, although the limited sample size did not allow for these differences to reach statistical significance [125]. This was accompanied by an increase in ileum excretion of fat and cholesterol. Dubois et al. observed that the addition of 10 g pea or soybean fibers to a high-fat meal did not result in a decrease in postprandial TG concentration in six healthy male adults but both fibers led to a decrease in CM-cholesterol and -phospholipid concentrations [126]. However, Sandstrom et al. showed that consumption of pea fibers by eight healthy adults in two consecutive meals (containing 7.4 g and 9.3 g fibers respectively) resulted in a reduction in CM-TG and intermediate-density lipoprotein-TG concentrations compared to a low-fiber meal [127]. The meals provided in this study had less fat (19 and 43 g), and hence more carbohydrates, than those used by Dubois et al. (70 g), which could partly explain the discrepancies between the two studies.

Kristensen et al. showed that addition of flaxseed fibers to a test meal containing 50 g fat led to a decrease in postprandial plasma TG concentration in 18 healthy male adults (with 22 < body mass index < 30 kg/m²) [128]. The difference (7 g dietary fibers) with the control meal was more marked with the meal that provided a high dose (17 g) of flaxseed fibers from mucilage than with the meals that provided 12 g dietary fibers from whole flaxseeds or low dose flaxseed mucilage. The addition of mucilage also led to a decrease in postprandial insulin secretion. Khossoussi et al. showed that addition of 12 g dietary fibers from psyllium husk to a standard meal (total fiber content = 15 g) led to a 21% decrease in postprandial serum TG concentration in 10 overweight and obese mean [129]. Moreover, apoB-48 concentration was lower 1h after consumption of the meal high in fibers compared to the meal low in fibers but the difference was not significant over a 6 h period. Resistant maltodextrin (5 or 10 g added to a test meal containing 50 g fat) has also been shown to elicit a decreased postprandial serum TG concentration in 13 healthy adults [130]. Kondo et al. provided 11 healthy male adults with moderate hypercholesterolemia 200 g yogurt with or without 6 g partially hydrolyzed guar gum together with a high-fat meal containing 43.5 g fat [131]. Ingestion of fibers led to a 15% decrease in postprandial serum TG concentration and a 23% decrease in postprandial remnant-like lipoprotein particle cholesterol concentration.

In contrast, some studies have shown no effect or even an increase in postprandial TG concentrations following acute consumption of fibers. For example, Bourdon et al. observed no effect of a barley pasta meal enriched with beta-glucan (15.7 g fibers versus 5 g fibers) on postprandial plasma or TRL-TG or -cholesterol concentrations or postprandial TRL-apoB-48 concentration in 11 healthy men [123]. Redard et al. observed a higher postprandial plasma TG concentration in females, but not in males, following consumption of a high-fiber (15.4 g mixture of oat bran and guar gum) versus a low-fiber (0.4 g) test meal [132]. Likewise, Ulmius et al. observed an increase in postprandial plasma TG concentration following consumption of meals enriched with fibers from oats, rye bran, sugar beet fibers or a mixture of these three fibers versus a low-fiber test meal in 13 healthy adults [133]. The meals providing fibers not only differed in their soluble fiber content but also in their insoluble fiber content and they were mixed with a blackcurrant beverage, hereby raising the question whether the way fibers are added to the food matrix can influence the postprandial response measured.

### 6.2. Effect of Chronic Consumption of Dietary Fibers

Maki et al. studied the effect of adding oat containing β-glucan or wheat cereal products to the usual diet of 27 healthy male adults for two weeks on their postprandial lipemia following consumption of a high-fat meal [134]. Both dietary treatments were matched for their energy and total fiber content. The postprandial serum peak TG concentration was lower and the postprandial serum TG concentration tended to be lower following consumption of the oat treatment but the postprandial FFA concentration was higher. Of note, Cara et al. did not observe any difference in postprandial serum-TG concentration following consumption of a meal containing 10 g β-glucan vs wheat fibers in healthy individuals [124]. Dubois et al. compared the postprandial effects of an oat bran test meal (12.8 g fibers) following consumption for 14 days of either an oat bran supplemented diet (23.8 g fibers/day) or a basal low-fiber diet (2.8 g fibers/day) in six normolipemic men [135]. Although it did not reach statistical significance, probably due to the low number of participants, the oat test meal following chronic consumption of oat elicited a slightly greater postprandial CM-TG concentration. However, the authors did observe significant differences in the postprandial concentrations of other lipid classes: postprandial plasma phospholipid, as well as plasma- and HDL-free cholesterol concentrations were increased while postprandial HDL-C ester concentration was decreased. The authors thus concluded that chronic consumption of oat exacerbates the acute effects of an oat meal on postprandial lipid concentrations. Wolever et al. provided 33 dyslipidemic participants for four months either a high soluble or insoluble fiber diet. The postprandial CM-TG concentration following consumption of a standardized fiber-free fatty liquid meal did not exhibit any difference when all subjects were considered but subjects with the APOε3 phenotype had a greater postprandial CM-TG concentration, due to an increase in the rate of fat absorption or CM synthesis or both, after soluble vs insoluble long-term fiber consumption [136]. Bozetto et al. provided 20 overweight/obese subjects with T2D with a high carbohydrate/fiber diet (26 g fibers/1000 kcal) for eight weeks and showed that participants exhibited a lower postprandial plasma TG concentration as well as a lower TG concentration in the CM+VLDL fraction following consumption of a test meal rich in saturated fat after the dietary intervention [137]. In another study using a relatively similar design, the same group also observed a decrease in postprandial CM-cholesterol concentration in T2D patients [138].

To summarize, most studies show that acute addition of soluble fibers to the meal leads to lower postprandial TG concentrations. Several interrelated mechanisms linked to lipid digestion have been implicated (see [121] for review). However, studies on this matter are relatively old and have thus not taken into consideration the possible effect of the interaction between the gut microbiota and fiber consumption on postprandial lipemia and more research is therefore needed to investigate if some strains of bacteria are associated with altered postprandial TG concentrations or lipoprotein profiles, as has been for example shown recently in the case of postprandial glucose concentrations [139].

## 7. Effect of Micronutrients and Phytochemicals 

Some micronutrients, e.g., vitamins or trace elements, as well as polyphenols have been shown to modulate postprandial lipemia. However, mechanisms underlying these effects have not been fully elucidated and it is possible that not yet studied vitamins or trace metals, e.g., fat-soluble vitamins or selenium, or phytochemicals other than polyphenols, e.g., carotenoids, might modulate postprandial lipemia as well.

### 7.1. Niacin

The term niacin (also known as vitamin B3) refers to all molecules with the biological activity of nicotinamide. It functions in the body as a component of Nicotinamide Adenine Dinucleotide Phosphate (NADP) and Nicotinamide Adenine Dinucleotide Phosphate hydrogen (NADPH), which are involved in many metabolic processes, including glycolysis, FA metabolism, and tissue respiration. Its effect on fasting blood lipids is well established and pharmacological doses of niacin have been used for five decades to treat lipid disorders and try to prevent CVD. Indeed, niacin diminishes fasting blood TG concentration, likely by its inhibitory effect on VLDL production in T2D patients [140], as well as LDL-C concentration, and raises HDL-C concentration in hypercholesterolemic patients and in subjects with diabetes and peripheral arterial disease [141,142]. Nevertheless, its role on postprandial lipemia is still controversial because the only 3 studies dedicated to this topic showed different results. Indeed, in a first study, niacin treatment did not significantly changed CM kinetics in subjects with isolated low HDL-C [143] while in a second one, which was performed in normolipidemic men with hypoalphalipoproteinemia, it significantly diminished it [144]. In a third study performed in T2D patients, an intermediate effect was observed with a decrease in the postprandial secretion rate of apoB-48-containing particles without a significant change in iAUC of postprandial plasma TG and apoB-48 concentrations [145]. The reason for this discrepancy is not known but it might be due to the form of niacin used, i.e., crystalline or another formulation, e.g., extended-release or sustained-release [146], as well as to the characteristics of the subjects. Although it has been shown, in lean and obese subjects, that niacin reduces FFA mobilization from adipocytes, perhaps by suppressing lipolysis [147], and diminishes the liver formation of TG via noncompetitive inhibition of liver diacylglycerol acyltransferase-2 (DGAT2) [148], mechanisms that explain its role on postprandial lipemia have not been elucidated until the results of a recent study that give an idea on the potential mechanism. Indeed, in a study, which was performed in statin-treated T2D subjects, it was observed that extended-release niacin reduced postprandial secretion rate of apoB-48-containing particles [145].

However, a recent meta-analysis of randomized controlled trial (39,195 subjects; median duration of treatment 11.5 months; median dose of niacin 2 g/day in monotherapy or in combination with other component versus placebo/usual care or other component alone) showed no reduction with niacin in mortality, cardiovascular mortality, fatal or non-fatal myocardial infarction nor fatal or non-fatal strokes but niacin was associated with side effects [149].

### 7.2. Zinc

The pioneering studies on the effect of Zinc (Zn) on postprandial lipemia in rats were published in 1977 by Koo and Turk [150,151]. These researchers described in detail the consequences of Zn deficiency on lipid absorption in the rat. They notably found that the rate of TG absorption markedly decreased, with a huge accumulation of TG droplets in the mucosa and that these droplets were unstable and coalesced. They also observed that cell cytoplasm exhibited prominent cellular changes. They deduced that the enterocytes were not able, by an unknown mechanism, to secrete lipid droplets. They suggested that this was due to the failure of these cells to synthesize proteins required for the formation of CM, i.e., apolipoproteins. Later, it was found that marginal Zn depletion significantly diminished apo-C and -E concentrations in CM [152]. The nascent CM were also irregular and larger in shape and size. The same team further showed in rats that the CM from marginally Zn-deficient rats were less efficiently taken up by the liver [153]. This likely explained their delayed clearance from the blood. One year later, the same team showed, also in rats, that marginal Zn deficiency also significantly diminished CM-apo-B concentration [154]. About ten years later, Reaves et al. showed that the plasma ratio of apoB-48 to total apoB protein was significantly lower in Zn-deficient rats than in Zn-adequate rats [155]. They suggested that this is due to the editing of apoBmRNA that was impaired by Zn deficiency. Indeed, apoB mRNA editing is performed by a Zn-containing cytidine deaminase [156] and this enzyme determines whether apoB-48 or apoB-100 is synthesized. The same team later focused on the intestine and found that Zn deficiency modestly, but significantly, diminished intestinal apoB mRNA editing in hamsters [157]. Nevertheless, another team did not observe significant modification of apoB mRNA editing in rats upon zinc deficiency [158]. This suggests that this effect, if existing, is not very important. Nevertheless, the demonstrated inhibitory effect of Zn deficiency on the intestinal synthesis of CM in rats and hamsters has also been observed in Mongolian Gerbils [159], suggesting that this is a general phenomenon in rodents and, we assume, in mammals. Unfortunately, to our knowledge, there is no data in human. Zn deficiency has apparently also another effect on CM metabolism via a reduction of their lipolysis efficiency by LPL. Indeed, Zn-deficient rats exhibited a reduced LPL activity in postheparin serum and adipose tissue [160,161]. Koo and Lee suggested that this was not due to changes in the enzyme activity per se, but to Zn-deficiency-induced compositional alterations in CM, which modulate LPL activity. The key role of CM composition on LPL activity likely explains why the effect of Zn deficiency on LPL activity was observed in rats fed coconut oil, but not in rats fed fish oil [161].

### 7.3. Copper

Although there are few studies on the effect of copper (Cu) on CM metabolism, the two available studies performed in rats suggest an effect of Cu deficiency. In the first study, it was observed that Cu deficiency significantly diminished the activities of both endothelial LPL and hepatic lipase. This might explain the lower clearance rate of CM, which was observed in the beginning of the postprandial period in the Cu deficient rats [162]. The second study showed that TRL isolated from Cu-deficient rats were more fluid than those isolated from control rats [163]. This was apparently due to their low cholesterol/phospholipid ratio and their high TG content. The authors suggested that these modifications could affect the metabolism of these lipoproteins. Thus, dedicated clinical studies are required to assess the effect of Cu deficiency on postprandial lipemia. 

### 7.4. Magnesium and Calcium

A pioneer study performed in inverted hamster intestine showed that a very low concentration of Mg in the intestinal lumen impairs the normal secretion of CM by the intestine [164]. Conversely, a clinical study performed in healthy individuals found that both the CM-TG response and the postprandial blood concentration of apoB-48 after a fat load were significantly lower after a meal that contained a Mg supplement (500 mg) than after a control meal [165]. Thus, as for Cu, further clinical studies are required to conclude on the effect of Mg status or Mg supplementation on postprandial lipemia. Concerning Calcium (Ca), a clinical study has suggested that dairy Ca, but not supplementary Ca carbonate, can attenuate postprandial lipemia in healthy moderately overweighted men [166]. It was suggested that this was due to impaired fat absorption because high Ca intake increases fecal fat excretion. However, further studies are needed to confirm this finding and to explain the different effect of these two chemical forms of Ca.

### 7.5. Polyphenols

The story on the effect of polyphenols on postprandial lipemia unusually started with a clinical study that found no significant effect of acute dealcoholized red wine, which is rich in polyphenols, on postprandial lipid metabolism in dyslipidemic postmenopausal women [167], suggesting that these polyphenols, at the tested dose, do not significantly affect lipid absorption and CM metabolism. However, a study performed in human Caco-2 cells led to an opposite conclusion by showing that red wine polyphenols significantly impaired the secretion of apoB-48 by these cells [168]. Conversely, in the same cell model, Vidal et al. did not find that wine polyphenols decreased the secretion of lipoproteins, contrarily to apple polyphenols that decreased it [169]. Tea polyphenols were also shown to decrease postprandial hypertriglyceridemia in rodents [170,171] and in men with mild or borderline hyperTG [172]. It was suggested that this was due to a decrease in TG absorption via an inhibition of pancreatic lipase activity [171]. However, as observed in mice, this could also be due to the fact that tea polyphenols decrease bile acid reabsorption, which results in lower intestinal bile acid levels, which might further decrease lipid absorption [173]. Interestingly, coffee polyphenols also inhibited pancreatic lipase activity, resulting in a lower postprandial increase in blood TG concentration. A study in mice suggested that this effect was apparently due mainly to one species of polyphenols among the 9 species that are recovered in coffee, i.e., di-cafeoylquinic acids [174]. Cinnamon extract, which is rich in polyphenols, was also able to diminish the secretion of apo-B48 and TRL in a fat load test performed in hamsters. Furthermore, it was observed that cinnamon extract reversed the expression of the impaired Insulin Receptor (IR), Insulin Receptor Substrate 1 (IRS1), IRS2 and AKT serine/threonin Kinase 1 (Akt1) mRNA levels and inhibited the overexpression of MTP and SREBP-1cin rodent enterocytes [175,176]. In another study, an anthocyanin-rich extract purified from a Haskap fruit significantly reduced the postprandial TG response measured in rats after a fat load [177]. Finally, a clinical study performed in overweight/obese subjects and components of the metabolic syndrome showed that subjects submitted to eight-week supplementation with a diet rich in polyphenols had lower postprandial TG response to a fat load than subjects who consumed a diet poor in polyphenols [178].

To summarize, it appears that some polyphenols, but not all, could significantly impair either the absorption of lipids or the intestinal secretion of CM. Nevertheless, additional studies, preferentially clinical ones, are required to identify which polyphenols and, at which dose, can significantly diminish postprandial lipemia.

## 8. Effect of the Food Structure (Matrix) 

Although the study of the effects of single nutrients on postprandial lipemia is paramount in our understanding of the mechanisms involved, this approach bears limitations since human beings consume foods and not isolated nutrients. Indeed, most foods are complex, heterogeneous matrices and are defined not only by their qualitative and quantitative molecular composition but also by the organization of their molecules at multiple spatial length scales [179]. Moreover, the initial structure of a food is greatly modified by digestive processes, be it physical (e.g., mastication, antral grinding) or chemical (e.g., digestive enzymes, pH) ones. Hence, numerous interactions exist between the different components of each food and with other components from co-consumed foods. Jenkins and colleagues have long acknowledged this complexity in the case of postprandial glycaemia with the introduction of the glycemic index in 1981, which considered the postprandial effect of both nutrients, such as mono- or di-saccharides, as well as that of foods [180]. To date, such an approach has not been developed in the case of postprandial TG although guidelines have been proposed to assess postprandial TG concentrations in a standardized fashion [181]. Yet, several authors have pointed at the greater efficacy of food-based approaches in the prevention and treatment of some chronic diseases, including CVD [182,183,184], and thus advocate to switch the focus from nutrients to foods, for easier translation to the public but also to take into account the inherent complexity of food matrices. Numerous studies have shown that the distribution of FA in TG, the organization of lipids as oil droplet emulsions differing in their size and interfacial composition, the degree of crystallized fat or the permeability of the food matrix to digestive enzymes can influence lipid digestion and metabolism [185] but only a few studies have specifically investigated the effects of food structure on postprandial lipemia.

### 8.1. Effect of Dietary Lipid Physical State

Fats and oils in foods can be present either as a continuous phase or as emulsions, i.e., two immiscible phases dispersed as droplets, but they are also characterized by their crystallized/liquid TG ratio, which varies with temperature. Vors et al. provided nine normal weight and nine obese subjects with an identical breakfast containing 40 g milk fat either emulsified or non-emulsified [186]. Importantly, the two fats used had similar melting temperatures. The emulsified fat led to an earlier and greater CM-TG peak concentration, greater apoB-48 concentrations in all subjects, as well as larger CM size and iAUC of the CM-TG concentrations in obese subjects. Garaiova et al. also observed a 60% greater iAUC of the postprandial plasma TG concentrations following consumption by 24 healthy volunteers of a standardized meal comprising 30ml of an emulsified *n*-3-rich PUFA (EPA + DHA = 28% *w/w*) oil mixture compared to the same meal but with a non-emulsified oil mixture [187]. Nevertheless, only the postprandial AUC for plasma *n*-3 PUFA concentrations was affected by the emulsification, i.e., AUC for postprandial plasma SFA, MUFA and *n*-6 PUFA concentrations were not significantly different, strongly suggesting an increase in the absorption efficiency of *n*-3 PUFA with emulsification rather than a modification of postprandial CM metabolism.

Clemente et al. investigated postprandial TG concentrations in 8 T2D overweight patients after they received three test meals, identical in volume and macronutrient composition, but with fat originating from different sources, namely milk, butter and mozzarella cheese. No significant difference was observed in the increase in plasma TG concentration over the 6 h following the meal although the meal containing butter elicited a significantly delayed plasma TG peak time, not due to differences in gastric emptying rate. Unfortunately, it is not possible to conclude from the study design if this was due to the dispersion state of lipids (relatively small native milk fat globules in milk, aggregated milk fat globules dispersed in a protein matrix for mozzarella cheese, relatively larger fat droplets for butter) or to differences in viscosity (butter, mozzarella cheese and milk being respectively solid, semi-solid and liquid) [188]. Tholstrup et al. did not observe either any difference in postprandial plasma total TG, CM-TG and VLDL-TG concentrations when they provided 14 healthy young men with butter, cheese and milk [189].

### 8.2. Effect of the Droplet Size of the Oil Emulsion

Fats and oils in processed foods are mostly found as emulsions, and usually as oil-in-water emulsions. The initial oil droplet size has a major impact on lipid digestion, with smaller droplet size leading to faster digestion rate due to increased surface area. Armand et al. fed 8 healthy individuals with either a fine (surface-weighted mean diameter = 0.7 µm) or a coarse (surface-weighted mean diameter = 10 µm) emulsion and they observed a higher gastric and duodenal lipolysis, a slower gastric emptying, confirmed in [190], and a later postprandial serum- and CM-TG peak concentrations with the fine emulsion, but no significant difference was observed in the AUC of the postprandial serum- or CM-TG concentrations [191]. Tan et al. also studied the effect of emulsification and oil droplet size on postprandial TG concentrations [192]. Fifteen healthy Chinese males received a test meal containing olive oil as non-emulsified, finely emulsified (surface-weighted mean diameter = 0.7 µm) or coarsely emulsified (surface-weighted mean diameter = 10 µm). The meal with non-emulsified oil elicited the lowest iAUC of the postprandial plasma TG concentrations (although only with a trend against the coarse emulsion, *p* = 0.07), associated with the fastest gastric emptying, in agreement with the above-mentioned results from Vors et al. [186]. Moreover, a higher iAUC of the postprandial plasma TG concentration was observed following consumption of the test meal containing the fine emulsion compared to that containing the coarse emulsion (similar surface-weighted mean diameters as in Armand et al.) [192].

### 8.3. Effect of the Interfacial Film at the Oil-in-Water Emulsion Droplet Surface

The formation and stability of oil-in-water emulsions in the gastro-intestinal tract is influenced by the presence of emulsifiers, such as proteins, polysaccharides or phospholipids, which can in turn modulate oil droplet coalescence and hence fat digestion rate. Proteins differ in their emulsifying and stabilizing capacities, depending partly on their solubility, their hydrolysis rate by proteases and their displacement from the interfacial film by bile salts (see [193] for review). These characteristics could explain some of the differences observed in studies comparing the effect of protein sources on postprandial TG concentration but this has usually not been evaluated, with the exception of Mariotti et al. who observed in vitro a phase separation with the casein meal used in their clinical intervention study which they suggest could partly explain the associated lower increase in postprandial TG concentration [116]. Keogh et al. fed 10 men and 10 women (mean age = 59 years) two emulsions (iso-viscous, iso-caloric and same mean droplet size), containing 30 g of fat, differing in their emulsifier composition (namely sodium sterol lactylate or sodium caseinate/monoglycerides) [194]. Emulsions stabilized by sodium caseinate/monoglycerides elicited lower postprandial TG concentrations at 90 and 120 min compared to sodium sterol lactylate (no AUC calculated), with a concomitant faster gastric emptying and lower secretion of the gut hormones cholecystokinin, GLP-1 and peptide YY.

### 8.4. Effect of the Positional Distribution of FA in TG

Dietary TG can vary in FA chain length, degree of unsaturation but also in the distribution of FA on the glycerol backbone (stereospecificity), whether in naturally occurring TG or from technological processing by food industries, a technique termed interesterification. The isomers thus formed can lead to TG molecules with different physical properties, including melting temperature [195], digestion rates and biological effects, including postprandial TG concentrations. Dedicated reviews on the effects of interesterification on lipid metabolism have previously been published [196,197]. Berry reviewed 10 cross-over studies investigating the postprandial effects of stearic and palmitic acid-rich fats, the two major SFA in human diets, where test meals only differed from control meals by the stereospecificity of the TG sources and not by the FA composition. No conclusion could be drawn when only the positional distribution of FA was considered but she suggested that interesterified TG with higher melting points, i.e., crystalline at body temperature, led to a decrease in postprandial TG concentration, due to a slower assembling of micelles, leading to a slower rate of lipolysis in the gastro-intestinal tract [196]. This hypothesis has been confirmed in two subsequent studies by Berry’s group where fat test meals containing interesterified palm olein led to lower postprandial plasma TG concentration compared to palm olein in healthy men and women [198] and in men aged 40–70 years with fasting plasma TG concentration > 1.2 mmol/L [199]. In these two studies, interesterified palm olein was characterized by a higher proportion of palmitic acid in the *sn*-2 position and a higher melting point (4.7% solid fat content at 37 °C whereas palm olein was fully melted at 37 °C). 

### 8.5. Effect of Fat Localization within the Food Matrix

Berry et al. showed that a test meal containing 54 g fat provided as whole almond seed macroparticles elicited a 74% lower postprandial increase in plasma TG concentration compared to the same test meal containing almond oil and defatted almond flour (identical macronutrient composition) in 20 healthy adult men [200]. Oils bodies in almonds, as in many nuts, are found within thin-walled cells. These cell walls have been found to be highly resistant to digestion since almond microstructure has been shown to be only marginally affected by mastication, leading to low lipid bioaccessibility and hence lipolysis rates [201].

### 8.6. Effect of the Meal Consistency/Viscosity

The effect of the addition of fibers on postprandial TG concentrations has been specifically addressed in the dedicated chapter and is therefore left out of this section.

In a recent study, Dias et al. investigated the effect of three meals differing in their structure and form, namely solid, semi-solid and liquid, while having the same nutrient composition, on lipid digestion and postprandial TG concentration using an *in vitro* approach and a randomized, cross-over, dietary intervention trial in 26 healthy adults [202]. They showed that the liquid food elicited significantly higher postprandial TG concentration compared to the solid food, while the semi-solid food displayed an intermediate figure though not reaching statistical significance. This effect was partly attributed to the larger oil droplet size exhibited by the solid food compared to semi-solid and liquid food before and in the earlier stages of in vitro digestions as well as to the fact that solid food showed phase separation during gastric digestion together with a lower release of FA during intestinal digestion.

To summarize, food structure, whether native or manipulated, can significantly affect postprandial TG concentrations and can even override the effects of macronutrient composition. Lipid emulsification, particularly with a smaller droplet size, interesterification leading to TG with lower melting points or lower meal viscosity all elicit higher postprandial TG. Additional research is warranted to better characterize how manipulation of food structure can impact on postprandial TG concentration, e.g., effects of emulsifier type on the stability of oil-in-water emulsions. Nonetheless, consumption of foods with specific food structure, e.g., nuts, or technological modification of food structure certainly constitute relevant approaches in the prevention and management of elevated postprandial TG concentrations.

## 9. Conclusions

During the last decades, many clinical studies have highlighted the fact that healthy humans spend most of their time in a hyperlipidemic postprandial state due to the repetitive consumption of fat-containing meals and that this process is exacerbated in hyperlipidemic patients. Postprandial lipemia is characterized by the accumulation of both hepatic apoB-100 and intestinally-derived apoB-48 TRL in the circulation, which participate in atherosclerotic plaque progression. Accordingly, postprandial lipemia, in both its magnitude and duration, has been shown to constitute an independent risk factor for CVD, which confirms the central role of dietary modifications in the treatment and prevention of CVD. 

Indeed, we have shown in this review that chylomicron production and postprandial lipemia are highly modulated by both habitual diet and single meal nutrient composition. Despite conflicting results between studies due to different methodological approaches and many potentially confounding factors, we have summarized in Table 1 the main acute and chronic effects of food components as well as food structure on chylomicron production and postprandial lipemia.

Postprandial lipemia increases dose-dependently with the amount of dietary fat or cholesterol after a single meal, over a certain-amount threshold. However, due to interactions between long-chain fatty acid species and physico-chemical properties of fat structures, especially fat droplet characteristics, it is difficult to identify the acute effect of FA species on postprandial lipemia. Despite some conflicting results, studies of habitual diet show postprandial lipidemic responses in the order SFA > MUFA > *n*-6 PUFA > *n*-3 PUFA.

Although dietary fat has received the most attention, other nutrients and micronutrients can modulate postprandial lipemia. Dietary proteins could apparently display some effect but it depends on their nature and the few studies available do not allow us to conclude on their effect on postprandial lipemia. Carbohydrate sources added to a meal have been shown to modulate postprandial lipemia in relation with their glycemic index. Added to a fat-meal, glucose, and more markedly fructose, can noticeably increase postprandial lipemia. Chronic hypercaloric intake of fructose but not isocaloric consumption of carbohydrates (fructose, glucose or starch) resulted in constant increase postprandial lipemia. Some minerals (Ca, Zn, Cu) and some polyphenols, e.g., tea polyphenols, have also been shown to modulate postprandial lipemia but the number of studies is limited and the mechanisms suggested deserve more investigations. Dietary fibers, especially soluble fibers from various origins, can lower postprandial lipemia when added to a fatty meal in sufficient amount. Mechanisms involved are not fully understood and new studies should thus be performed to evaluate the possible interaction between the gut microbiota with the effect of fiber consumption on postprandial lipemia. Finally, nutrient composition alone cannot explain the effect of foods on postprandial lipemia and it is now clear that the food matrix is a key factor influencing fat digestion and hence postprandial lipemia. 

The potential mechanisms of action have been reviewed and summarized in Table 2 but are not fully understood. Further studies and particularly lipoprotein kinetic studies in humans are needed.

Dietary approaches based on food nutrient composition and structural interactions represent relevant approaches to control postprandial lipemia.

A better understanding of the factors and mechanisms regulating chylomicron production and postprandial lipemia, and particularly diet, is essential to try to modulate their increases and thus reduce the risk of atherosclerotic cardiovascular diseases and potentially the risk of total mortality.

## Figures and Tables

**Table 1:** Effects of nutrients and micronutrients on postprandial lipemia.

Dietary Components		Postprandial Lipemia	Level of Evidence
Fats	Amount	↑	+++
	Type (acute)	SFA = MUFA = PUFA	++
	Type (chronic)	SFA > MUFA > *n*-6 PUFA > *n*-3 PUFA	++
	Amount of cholesterol	↑	++
Carbohydrates	Acute	↑ (fructose > glucose)	+++
		↑ (glycemic index)	+++
	Chronic	↑ (fructose/dose dependent)	+++
Proteins	Whey proteins (acute)	↓	++
Fibers	Soluble (acute)	↓	++
Micronutrients	Ca supplement	↓	+
	Niacin supplement	↓	++
	Zn deficiency	↓	+
	Cu deficiency	↑	+
	Mg supplement	↓	+
	Polyphenol supplement	↓	+

Ca, calcium; Cu, copper; Mg, magnesium; MUFA, monounsaturated fatty acids; PUFA, polyunsaturated fatty acids; SFA, saturated fatty acids; Zn, zinc; +++: convincing; ++: probable; +: suggested.

**Table 2:** Potential mechanisms of nutrients and micronutrients action in postprandial lipemia.

Dietary Components		Potential Mechanisms
Fats	MUFA	↓ PR and ↑ FCR VLDL
	*n*-6 PUFA	↑ FCR VLDL
	*n*-3 PUFA	↓ PR CM; ↓ PR and ↑ FCR VLDL
Carbohydrates	Fructose	↑ PR CM; ↑ PR VLDL
	Glucose	↑ PR CM
Proteins	Whey protein	Slowed gastric emptying ↑ insulin release ↓ PR CM
Fibers		Slowed gastric emptying Alteration of TG hydrolysis Alteration of mixed micelle formation ↓ PR CM
Micronutrients	Ca supplement	↓ fat absorption
	Niacin supplement	↓ PR CM
	Zn deficiency	↓ TG absorption rate and PR CM
	Cu deficiency	↓ LPL and HL activities
	Mg supplement	↓ PR CM
	Polyphenol supplement	↓ TG absorption likely by ↓ pancreatic lipase activity

Ca, calcium; Cu, copper; Mg, magnesium; CM, chylomicrons; FCR, fractional catabolic rate; MUFA, monounsaturated fatty acids; PR, production rate; PUFA, polyunsaturated fatty acids; VLDL, very low, density lipoprotein; Zn, zinc.
